# Notes on Hospital Planning

**Published:** 1911-02-18

**Authors:** A. Saxon Snell

**Affiliations:** 22 Southampton Buildings, Chancery Lane, W.C.


### Institutional Letter-Box.

### NOTES ON HOSPITAL PLANNING.

*To the Editor of* The Hospital.

Sir,—The publishers have been kind enough to send me a marked copy of the current issue of The Hospital (though, by the way, I am a subscriber), drawing my attention to the Notes on Hospital Planning by Dr. Goldwater. It is at all times interesting and instructive to study other people's and other countries' views upon hospital planning.

The author claims some originality for the plan illustrated. Is it not rather reversion to an old and abandoned type? Economy in space is no doubt a good thing, especially for city sites; but it seems scarcely worth so much sacrifice of essential principles. One end of the long ward would be badly lighted and ventilated. Four of the separation wards face the main street and have north lighting only. The sanitary conveniences are wedged in between rooms without cross-ventilation. Is it possible too that the small closets at the end of the long ward and opening direct into it are fitted with sinks?—Yours faithfully,

22 Southampton Buildings, Chancery Lane, W.C. — A. Saxon Snell.

"Paris."—Removal of tattoo marks is possible but the process is a very long and complicated one of which we cannot give details here. A skin specialist will be able to advise you.

"R. F. P."—You are quite wrong. The London Hospital is by no means the largest institution of its kind in the world. The largest hospitals are the Virchow at Berlin and the Maggiore at Milan. The London has the distinction of being the largest voluntary hospital in the British Empire.

"Practitioner."—There is no such book published as yet, and we will keep the suggestion in mind. Write to the Oxford Medical Publication (Hodder and Stoughton, Warwick Square, E.C.) for a list of their manuals suitable for general practitioners.

"Enquirer."—"Burdett's Hospitals and Charities" is the standard work on the subject so far as the English-speaking world is concerned. You will find in it full details about the Canadian and Newfoundland hospitals.

